# Supplementary material for: A fault-intrusion-tolerant system and deadline-aware algorithm for scheduling scientific workflow in the cloud
Source: PeerJ Comput Sci. 2021 Nov 2;7:e747. doi: 10.7717/peerj-cs.747 (PMC8576554; doi:10.7717/peerj-cs.747)
Supplement: Supplemental Information 6 [file peerj-cs-07-747-s006.zip › Myproject1.1/docs/org/workflowsim/clustering/balancing/metrics/DistanceVariance.html]

DistanceVariance


JavaScript is disabled on your browser.


- Overview
- Package
- Class
- Use
- Tree
- Deprecated
- Index
- Help

- Prev Class
- Next Class

- Frames
- No Frames

- All Classes

- Summary:
- Nested |
- Field |
- Constr |
- Method

- Detail:
- Field |
- Constr |
- Method


org.workflowsim.clustering.balancing.metrics

## Class DistanceVariance

- java.lang.Object
- - org.workflowsim.clustering.balancing.metrics.DistanceVariance

- All Implemented Interfaces:
  :   BalancingMetric

  ---

    

  ```
  public class DistanceVariance
  extends java.lang.Object
  implements BalancingMetric
  ```

  A DistanceVariance

  Since:
  :   WorkflowSim Toolkit 1.0

- - ### Constructor Summary

    Constructors

    Constructor and Description

    `DistanceVariance()`
  - ### Method Summary

    Methods

    | Modifier and Type | Method and Description |
    | --- | --- |
    | `double` | `getMetric(java.util.ArrayList<TaskSet> list)` Returns the distance variance of a list of taskSets |

    - ### Methods inherited from class java.lang.Object

      `clone, equals, finalize, getClass, hashCode, notify, notifyAll, toString, wait, wait, wait`

- - ### Constructor Detail


    - #### DistanceVariance

      ```
      public DistanceVariance()
      ```
  - ### Method Detail


    - #### getMetric

      ```
      public double getMetric(java.util.ArrayList<TaskSet> list)
      ```

      Returns the distance variance of a list of taskSets

      **Specified by:**
      :   `getMetric` in interface `BalancingMetric`

      Parameters:
      :   `list` - the list to be checked

      Returns:
      :   the distance variance


- Overview
- Package
- Class
- Use
- Tree
- Deprecated
- Index
- Help

- Prev Class
- Next Class

- Frames
- No Frames

- All Classes

- Summary:
- Nested |
- Field |
- Constr |
- Method

- Detail:
- Field |
- Constr |
- Method
